# Quantitative Biomedical Imaging: Techniques and Clinical Applications

**DOI:** 10.1155/2016/3080965

**Published:** 2016-07-25

**Authors:** Guang Jia, Steven B. Heymsfield, Jinyuan Zhou, Guang Yang, Yukihisa Takayama

**Affiliations:** ^1^Medical and Health Physics Program, Department of Physics and Astronomy, Louisiana State University, Baton Rouge, LA 70803, USA; ^2^Laboratory of Metabolism-Body Composition, Pennington Biomedical Research Center, Baton Rouge, LA 70808, USA; ^3^Department of Radiology, Johns Hopkins University, Baltimore, MD 21287, USA; ^4^Shanghai Key Laboratory of Magnetic Resonance, Department of Physics, East China Normal University, Shanghai 200062, China; ^5^Department of Radiology Informatics and Network, Graduate School of Medical Sciences, Kyushu University, Fukuoka 812-8582, Japan

For decades, cross-sectional biomedical images have been generated from various modalities, including computed tomography (CT), three-dimensional tomosynthesis, ultrasound, magnetic resonance imaging (MRI), single photon emission computed tomography (SPECT), and positron emission tomography (PET). Many advanced quantitative imaging methods have been developed, such as perfusion MRI/CT, diffusion tensor/weighted MRI, functional MRI (fMRI), ultrasound/MR elastography, dynamic PET, and dynamic contrast enhanced MRI. There is great variability across imaging platforms, imaging techniques, postprocessing software, and imaging readers. There is an unmet clinical need for improving the value and practicality of quantitative biomedical imaging.

Systematic reviews of a quantitative biomedical imaging method will improve researchers' understanding and skills in utilizing these methods. For example, arterial spin labeling (ASL) is a noninvasive MRI modality capable of measuring blood perfusion without the use of a contrast agent. E. Vaghefi and B. Pontré reviewed the technical aspects of ASL and their implications for its optimum adaptation for retinal blood perfusion monitoring, as well as ASL application in human ocular blood flow assessment.

Cross-system/site/vendor/platform/software/reader comparisons of a quantitative biomedical imaging method are crucial. C. Brodén et al. compared 3D-CT to standard radiostereometric analysis for measuring migration of acetabular cups in total hip arthroplasty. G. J. Pelgrim et al. evaluated the capability of MRI and CT to perform myocardial perfusion quantification, previously only achievable with PET.

Reproducibility and reliability assessments of quantitative biomedical imaging methods are necessary steps. The current 2D tagging cardiac MRI technique requires multiple breath holds to cover the whole heart and cannot show the 3D motion of the left ventricle. Y. Amano et al. evaluated the feasibility of fast 3-breath-hold 3D tagging for the assessment of the circumferential strain in patients with hypertrophic myocardial diseases. A. R. Yu et al. investigated the optimal PET energy window for 124I PET based on image characteristics of reconstructed PET. The energy window of 350~750 keV was proposed as the optimal energy window, although 400~590 keV was obtained as the highest noise equivalent count rate.

Pathophysiological validation and computer simulation are crucial to understanding a quantitative biomedical imaging method. U. Klose et al. confirmed the strong dependence of the whole brain apparent diffusion coefficient (ADC) MRI histograms on the age of the examined subjects. The proposed model can be used to characterize changes of the whole brain ADC histogram in certain diseases under consideration of age effects. Z. Krajnc et al. quantitatively evaluated growth plates around the knees in adolescent soccer players utilizing the diffusion-weighted MRI. Diffusion-weighted imaging measurements indicate increased cellularity in growth plates around knees in football players most prominent in proximal tibia medial region after intense training. D. Shimamoto et al. evaluated whether the diagnostic performance of Gd-EOB-DTPA-enhanced MRI in evaluating liver function and pathology is improved by considering liver volume. They found that the inclusion of liver volume may improve Gd-EOB-DTPA-based predictions of liver function but not predictions of liver pathology. F. F. Schröder et al. analyzed preoperative CT images of patients who underwent pancreatoduodenectomy (PD) or pylorus preserving PD and investigated predictors for postoperative pancreatic fistula and postoperative severe complications.

Physical and virtual phantom for quality check/assurance will improve our knowledge of a quantitative biomedical imaging method. Characterization of lesion formation and restoration by imaging features is a novel field of research in multiple sclerosis. R. K. Verma et al. investigated statistical differences with MR perfusion imaging features that reflect the dynamics of Gadolinium uptake in multiple sclerosis lesions using dynamic texture parameter analysis. Brain tissue segmentation in MRI is useful for a wide range of applications. F. Baselice et al. proposed a brain joint segmentation and classification algorithm based on proton density and relaxation times, instead of the acquired gray level image.

Computer assisted analysis and diagnosis will facilitate the clinical adoption of single or multiparametric/modality imaging methods. In order to accurately diagnose acute appendicitis, K. B. Kim et al. proposed a method to extract the appendix automatically by using a series of image processing and self-organizing maps that learn typical shape patterns of the appendix from US. The analysis and interpretation of high-resolution CT images of the chest in the presence of interstitial lung disease are a time-consuming task which requires experience. V. Vasconcelos et al. proposed a computer-aided diagnosis (CAD) scheme to assist radiologists in the differentiation of lung patterns associated with interstitial lung disease and with healthy lung parenchyma. Indirect immunofluorescence is the gold standard for the diagnosis of autoimmune diseases. A. B. Elgaaied et al. introduced the AIDA Project (Autoimmunity: Diagnosis Assisted by Computer) developed in the framework of an Italy-Tunisia, cross-border cooperation and its preliminary results. J. Jeong et al. proposed a CAD algorithm with a simplified false-positive reduction scheme for microcalcification clusters in reconstructed digital breast tomosynthesis images. M. Larobina et al. investigated the feasibility of automatically training supervised methods, such as *k*-nearest neighbor and principal component discriminant analysis, to segment the four subcortical brain structures: caudate, thalamus, pallidum, and putamen. Their results demonstrate that atlas-guided training is an effective way to automatically define a representative and reliable training dataset, thus giving supervised methods the chance to successfully segment brain MRI images without the need for user interaction.

Development of a translational imaging method from preclinical to clinical patient care may require additional steps to simplify the paradigm, to improve image quality, or to reengineer the hardware. The usefulness of ADC MRI as a quantitative imaging tool has motivated several studies that have investigated the reliability and reproducibility of ADC estimates. M. Alipoor et al. proposed a new experiment design method that is based on minimizing the determinant of the covariance matrix of the estimated parameters. S. Aootaphao et al. proposed the X-ray scattering correction method for improving soft tissue images on the large flat panel detector of portable cone beam CT (CBCT). The reconstructed images with their proposed scatter correction show significant improvement on image quality. Thus, the proposed scatter correction technique has a high potential to detect soft tissues in the brain. S.-S. Han et al. evaluated the availability of software-based correction of mandibular plane for the vertical measurement of the mandible in CBCT. G. Wang et al. developed high-field permanent magnetic circuit of 1.2 T and 1.5 T with novel magnetic focusing and curved surface correction. They have obtained high quality images of mice using their small animal micro-MRI instruments.

Imaging-based big data and network systems may be used to promote hardware and software standards in quantitative biomedical imaging. Using the data from the Osteoarthritis Initiative, M. Zhang et al. developed a rapid cartilage damage quantification method for the lateral tibiofemoral compartment using MRI. M. Zhang et al. evaluated the influence of MRI sequence on the relationship between bone marrow lesions volume and pain. They compared quantitative assessments of bone marrow lesions on intermediate-weighted fat suppressed (IW FS) turbo spin echo and 3-dimensional dual echo steady state (3D DESS) sequences. They found that bone marrow lesion quantification on IW FS offers better validity and statistical power than bone marrow lesion quantification on a 3D DESS sequence.

Finally, it is crucial to develop novel quantitative biomedical imaging biomarkers for diseases, such as shear-wave ultrasound elastography (SWE). SWE is thought to be useful for quantitatively evaluating tissue hardness. However, it remains unclear what types of pathology affect tissue hardness. T. Fukuhara et al. elucidated the correlation between shear-wave velocity (SWV) and fibrosis in thyroid. Small muscle cells of the cavernosum play an important role in erection. J.-J. Zhang et al. investigated the feasibility of shear-wave US elastography on evaluating the level of small muscle cells in penis quantitatively. Liver disease associated with cystic fibrosis (CFLD) is the second cause of mortality in these patients. T. Cañas et al. found that shear-wave elastography in the right hepatic lobe is a noninvasive technique useful to detect CFLD in their sample of patients. They found that splenic SWV values are higher in cystic fibrosis patients, without any clinical consequence. Last but not least, V. Rajagopalan and E. P. Pioro studied the potential role of brain parenchymal fraction as a relatively simple quantitative MRI measure for distinguishing amyotrophic lateral sclerosis phenotypes.

*Guang Jia* *Steven B. Heymsfield* *Jinyuan Zhou* *Guang Yang* *Yukihisa Takayama*

